# The Relation Between Osteoporosis and Bone Fractures and Health-Related Quality of Life in Post-menopausal Saudi Women in the Jazan Region: A Cross-Sectional Study

**DOI:** 10.7759/cureus.54412

**Published:** 2024-02-18

**Authors:** Maged El-Setouhy, Zenat Khired, Hussam Darraj, Basem Zogel, Mohammed H Alhazmi, Rawan E Maghrabi, Maram Sayegh, Ahmed A Akkur, Nawaf Bakri, Asma Alhazmi, Mohammad Zaino

**Affiliations:** 1 Family and Community Medicine, Faculty of Medicine, Jazan University, Jazan, SAU; 2 Community, Environmental and Occupational Medicine, Faculty of Medicine, Ain Shams University, Cairo, EGY; 3 Surgery, Faculty of Medicine, Jazan University, Jazan, SAU; 4 Medicine and Surgery, Faculty of Medicine, Jazan University, Jazan, SAU; 5 Medicine and Surgery, Jazan University, Jazan, SAU; 6 Physical Therapy, Faculty of Applied Medical Science, Jazan University, Jazan, SAU

**Keywords:** environmental functioning, social functioning, psychological functioning, physical functioning, postmenopausal women, hrqol, fragility fracture, osteoporosis

## Abstract

Introduction: Osteoporosis is a significant health concern, often leading to fragility fractures and severely impacting the quality of life in post-menopausal women. Studies evaluating the effects of osteoporosis and resultant fractures on health-related quality of life (HRQoL) in Saudi women are lacking. This study aimed to assess the relationship between osteoporosis and fracture and physical, psychological, social, and environmental HRQoL domains in post-menopausal Saudi women.

Methods: In this cross-sectional study conducted in Jazan, Saudi Arabia, 158 post-menopausal Saudi women completed HRQoL surveys using the validated Arabic WHOQOL-BREF questionnaire. Data on socioeconomics, comorbidities, and fracture history were gathered. Descriptive statistics delineated sample characteristics. Analysis of variance (ANOVA) and post-hoc tests identified differences in HRQoL across socioeconomic and clinical categories. Multivariate regression analyses determined factors independently related to HRQoL.

Results: Of 158 women surveyed, 39% had a history of osteoporotic fracture. Foot (35%), hand (31%), and vertebral (10%) fractures were the most frequent. Women over 70 had significantly lower physical HRQoL than those aged 45-55 (p<0.001). Unemployed and lower-income women showed poorer HRQoL across domains (p<0.01). Vertebral and hand fractures were negatively related to physical and psychological health (p<0.05). Chronic diseases like hypertension and rheumatoid arthritis reduced HRQoL (p<0.01). In regression analyses, older age, vertebral fracture, physical inactivity, long-term hormone therapy, and unemployment emerged as determinants of poorer HRQoL (p<0.05).

Conclusion: Osteoporosis and resultant fragility fractures, especially in vertebral and hand bones, led to substantial impairments in physical, social, psychological, and environmental HRQoL in Saudi women. Modifiable risk factors like physical inactivity and long-term hormone use also affected HRQoL. Targeted screening and multidomain interventions for disadvantaged women with osteoporosis are warranted to improve functioning and quality of life.

## Introduction

Osteoporosis is characterized by low bone mineral density (BMD) resulting from an altered bone microstructure, making patients susceptible to low-impact fragility fractures. Increased morbidity, mortality, and disability resulting from osteoporotic fractures lead to a severe deterioration in quality of life [1]. Additionally, they impose a considerable economic burden on society regarding the direct use of healthcare resources such as hospitalizations, outpatient visits, prescription medication, rehabilitation, and long-term care, as well as direct non-medical expenses like transportation costs and the hiring of caregivers. Moreover, indirect costs are to be considered, such as the impact on family caregivers' time and work, the loss of patient productivity, and the potential for early retirement [2-7]. Osteoporosis showed the most vital link with distal radius fractures, pathologic vertebral fractures, lumbar and thoracic vertebral fractures, and femoral neck fractures across all risk factor groups [7,8].

Osteoporosis is a global health concern that has affected many people worldwide, with estimates indicating that more than 200 million individuals are affected [9]. The condition is expected to continue rising in prevalence due to the aging population [10]. An estimated 3.79 million osteoporotic fractures were recorded across the European Union in 2000. Of patients who experience a baseline fracture, 20% have a second fracture within the first year, suggesting this is a significant predictor of future fractures. Post-menopausal Jordanian women had an osteoporosis prevalence of 19.8% compared to post-menopausal Saudi Arabian women, who had a prevalence of 63.63% [11,12].

Low bone mass and rapid bone loss are independent risk factors for post-menopausal osteoporosis in women with a low body weight, a low body fat percentage, or a low body mass index (BMI) [9]. Both low serum total estradiol concentrations (<5 pg/mL) and high serum sex hormone-binding globulin concentrations (≥1 μg/dL) have been linked to an increased risk of hip and vertebral fractures in women 65 years of age and older, independent of BMD [13,14].

For all post-menopausal women, it is appropriate to evaluate these risk factors to determine who should receive an osteoporosis screening and to suggest nonpharmacologic measures like a balanced diet-especially for sufficient levels of protein, calcium, and vitamin D-regular exercise, and to avoid smoking and consuming alcohol excessively. To prevent bone loss, estrogen or other therapy options are available to women at high-risk for osteoporosis, particularly perimenopausal women with low bone density and other risk factors [9]. The main objective of treatment for women with osteoporosis and other fracture risk factors, such as advanced age and prior fractures, is to avoid more fractures. This is achieved by combining nonpharmacologic management, medications to promote bone strength and density, and strategies for preventing falls. If pharmacologic management is required, the government has approved choices, including estrogen agonists and antagonists, bisphosphonates, RANK ligand inhibitors, parathyroid hormone-receptor agonists, and sclerostin inhibitors [15]. This study evaluates the relationship between osteoporosis and fracture and health-related quality of life (HRQoL) in post-menopausal Saudi women.

## Materials and methods

We conducted this study using a cross-sectional design in the Jazan region between February and June 2023, located in the southwest of Saudi Arabia. The province is home to approximately 1.7 million people. We included a sample of post-menopausal Saudi women in this study to evaluate the relation between osteoporosis and fracture and HRQoL in post-menopausal Saudi women at the time of the study, and those who refused to participate were excluded.

The total sample of post-menopausal Saudi women in the Jazan region is about 385, sufficient to give a 95% confidence interval with a 5% margin of error. The sample size for this study was calculated using the Raosoft sample size calculator (Raosoft Inc., Seattle, WA, USA, raosoft.com).

We randomly selected five primary health care centers, namely, South Abuarish, South Damad, Ahad Al-Masarihah, Mokhatat 5, and Aljabal in Jazan were searched for post-menopausal women with osteoporosis and bone fractures who had visited the clinic or were in the ward. Next, the participants were contacted and scheduled for an interview to be conducted by the researchers. The participants were asked to answer the researchers' questionnaire during the interview. This study collected the data using an Arabic-language questionnaire. The questionnaire contains four sections. The first part of the questionnaire starts with socio-demographic factors such as age, gender, nationality, marital status, specialty, and income. The second part was developed to collect personal characteristics such as BMI, chronic diseases, smoking, and some risk factors. The third part is concerned with the history of osteoporosis, such as age when diagnosed with osteoporosis, how long the patient has been diagnosed, bone density, and how the patient treats osteoporosis. The fourth part was the WHOQOL-BREF questionnaire, which is made of 26 questions to collect data about the quality of life.

Before starting the information-gathering step, a pilot study was applied among 15 post-menopausal Saudi women who were not a part of this study to assess whether the questionnaire items were clear and understandable and to determine the time needed for the interview.

Data were analyzed using the statistical software SPSS version 24 (SPSS Inc., Chicago, IL, USA). Descriptive analysis was used to describe the data, and statistical analyses such as chi-square and regression were used to analyze the categorical variables. The numeric variables were analyzed using the t-test and the ANOVA test to test the mean differences for variables with two or more categories. Multilevel linear regression analysis was used to test the demographic characteristic's adjusted effect on the dependent studied variable, HRQOL. Frequency and prevalence data were presented in percentages (%). A p-value ≤ 0.05 was considered statistically significant.

Ethical considerations

Ethical approval to conduct this study was obtained from the Scientific Research Ethics Committee at the Ministry of Health in Saudi Arabia with No. 2314 on 06/02/2023. Informed consent was obtained from all participants. Privacy and confidentiality were preserved, and no participants were asked about anything that could reveal their identity or personal information.

## Results

The total number of women in the study was 158, with a mean age of about 53 years. The participants' baseline data showed that 62 women (39%) had osteoporosis and an incident fracture during their lives, and 96 women (61%) had osteoporosis without a fracture.

This study examined the locations of fractures in 62 women with osteoporosis. As shown in Table 1, the most common fracture site was the foot, accounting for 35% of cases (n = 22). Nearly one-third of fractures occurred in the hand (31%, n = 19). Fractures of the femur and vertebral column represented 11% and 10% of cases, respectively (n = 7 and n = 6). Pelvic fractures occurred in 8% of patients (n = 5). The least common fracture site was the hip, which occurred in only 5% of the sample (n = 3). Lower extremity fractures distal to the knee, including feet and hands, predominated in this cohort of women with osteoporosis, representing 66% of fractures (n = 41). Proximal femur and vertebral fractures accounted for 21% of cases (n = 13). These results provide insights into the anatomical distribution of osteoporotic fractures in this population, with hand, foot, and vertebral fractures being the most prevalent.

**Table 1:** Fracture location in women with osteoporosis.

Location of the fracture	n	%
Foot	22	35
Hip	3	5
Femur	7	11
Pelvis	5	8
Vertebral column	6	10
Hand	19	31

Table 2 presents the relationship between socio-demographic factors and HRQoL domains in women with osteoporosis. A one-way ANOVA identified significant differences across categories. Physical health scores were lower in those over 70 versus those aged 45-55 (p<0.001). Employed women had a higher psychological, social, and environmental quality of life than homemakers (p<0.001). Women with family incomes above 20,000 SAR had higher environmental domain scores than lower-income groups (p<0.001). A healthy BMI was associated with better social relations than an underweight BMI (p<0.001). Women with vertebral fractures and hand fractures had a poorer relation to physical, psychological, and social health versus no fractures. Married women had lower social relations scores than single women. Advanced age, vertebral and hand fractures, a low socioeconomic status, unemployment, an unhealthy BMI, and negatively impacted quality of life indicate high-risk subgroups in women with osteoporosis who may benefit from supportive interventions. Marital status and smoking also showed selective relations with the quality of life.

**Table 2:** The relationship between demographic variables and the HRQoL of the studied population. Values are presented as mean value±standard deviation (t-test, one-way ANOVA, and Bonferroni post-hoc test). The superscript letters A, B, C, D, and E mean a significant result with the corresponding category P<0.001. Larger values represent better HRQoL and BMI. ANOVA: analysis of variance, HRQoL: health-related quality of life, BMI: body mass index.

Socio-demographic characteristics	Category	Physical health	Psychological health	Social relations	Environment
		Mean±SD	Mean±SD	Mean±SD	Mean±SD
Location of fracture	No fracture^A^	64.6±14.9	66.8±12.7	69.2±19.9	64.5±12.4
	Foot^B^	65.5±17.4	73.1±16.9	73±20	66.3±15.9
	Hip, femur, pelvis^C^	61.4±15.4	63.7±15.8	70±18.9	63.3±15.1
	vertebral column^D^	51.5±15.5	55.5±14.4	51±18.4	57.3±13.8
	Hand^E^	56.2±15.1	59.9±13	66.8±12.6	59.7±8.8
Age category	45-55^A^	65.2±14.3^C^	68.1±14.1	68.8±19.7	65.4±12.5
	56-70^B^	59.7±16.5	62.5±13.3	70.6±16	60.3±12.3
	>70^C^	44.3±17.8	58.3±14.5	56.3±29.1	59.7±20.1
Employment status	Housewife^A^	60±15.1	61.4±15.2	61.2±25.6	59±15.2
	Working^B^	64.1±15.9	70.3±12.2^A^	75.2±12.6^A^	67.5±12.3^A^
	Retired^C^	64.2±15.7	66.7±13.8	69.9±15.3	64.7±10.1
Marital status	Single^A^	62.4±14.6	69.3±13.4	73.5±15.1	67.9±12.7
	Married^B^	63.8±15.3	65.9±15	67.7±20.1	63.4±13.2
	Divorced^C^	65.7±10.3	71±18.9	70±2.4	67.7±9.3
	Widow^D^	59.6±18.2	63.7±10.3	68.7±20.8	61.4±12.5
Family income	<3000^A^	57.5±11.6	58±12.8	60.2±23.1	54.2±9.4
	3000-5000^B^	63.7±15.4	65.8±15.6	73.5±14.7	64.3±16.1
	5001-10,000^C^	64.2±13.7	65.1±15.1	63.2±24.9	63.5±12.4
	10,001-20,000^D^	62.4±16.9	67±12.3	72.7±14	64.1±11.2
	>20,000^E^	63.8±20.6	75.8±15.7	65.3±19.6	73±15.4^E^
BMI	Underweight^A^	57.4±17.2	60.2±13.1	55.6±29.4	61.5±14.6
	Healthy weight^B^	63.8±15.2	67.3±13.9	71.6±16.3^A^	64±13
	Overweight^C^	63±14.1	65.3±14.2	67±14.6	64±10
	Obesity^D^	69±35.4	78±31.1	84.5±21.9	75±26.9
Smoking status	No^A^	62.9±15.8	65.7±14.1	68.5±19.8	63.4±12.9
	Yes^B^	62.8±13.1	71.4±14	73±8.6	68.8±12.7

These findings suggest that these factors can identify high-risk subgroups who may benefit from supportive interventions. Additionally, the relationship between smoking and quality of life was also noted. These results highlight the importance of considering socio-demographic factors when assessing and addressing HRQoL in women with osteoporosis.

Table 3 displays the relationship between comorbidities and quality-of-life domains in women with osteoporosis. Those with chronic diseases showed significantly lower physical health versus those without chronic conditions (p<0.001). Hypertension was associated with poorer social relationships (p<0.001). Rheumatoid arthritis (RA) had a negative effect on physical health compared to counterparts without it (p<0.001). Malabsorption syndrome corresponded to lower social functioning (p<0.001). Vitamin D deficiency and lack of exercise were linked to poorer scores across most domains (p<0.001). Long-term hormone therapy is related to lower psychological and social health (p<0.001). Diabetes, early menopause, hyperthyroidism, visual impairment, and previous osteoporotic fractures did not have statistically significant relations. In summary, chronic diseases such as hypertension, RA, malabsorption syndrome, vitamin D deficiency, and a sedentary lifestyle negatively affect the quality of life in women with osteoporosis. These findings highlight the importance of managing comorbidities and promoting healthy behaviors to improve the well-being of individuals with osteoporosis.

**Table 3:** The relation of comorbidity and HRQoL of the studied population. Values are presented as mean value±standard deviation (t-test, one-way ANOVA, and Bonferroni post-hoc test). Superscript letters A and B mean a significant result with the corresponding category P<0.001. Larger values represent better HRQoL and BMI. ANOVA: analysis of variance, HRQoL: health-related quality of life, BMI: body mass index.

Comorbidity	Category	Physical health	Psychological health	Social relations	Environment
		Mean±SD	Mean±SD	Mean±SD	Mean±SD
Chronic diseases	No^A^	66±14.5^B^	66.7±12.7	70.7±18.6	65.4±12.8
	Yes^B^	59.9±16	65.6±15.5	67±19.7	62.3±12.9
Hypertension	No^A^	63.9±15.6	66.8±13.2	71.1±16.7^B^	64.6±12.2
	Yes^B^	60.6±15.5	64.6±16.1	63.6±23.3	61.9±14.3
Rheumatoid arthritis	No^A^	64.2±15.5 ^B^	66.8±14.3	69.5±18.5	64.1±13.2
	Yes^B^	51.8±11.7	60.3±11.7	63.5±24.4	60.9±10.5
Hyperthyroidism	No^A^	62.9±15.5	66±14.1	68.7±19.5	63.8±13
	Yes^B^	62.8±18.8	69.5±16.3	71±13.9	62.8±12.4
Malabsorption syndrome	No^A^	62.9±15.8	65.9±14.3	69.6±18.5 ^B^	64±12.9
	Yes^B^	61.6±10.3	72.4±9.5	44.8±26.7	57.8±12
Type 1, 2 diabetes mellitus	No^A^	62.7±16	65.8±14.1	68.3±20.7	63.7±13.4
	Yes^B^	63.4±14.3	66.9±13.6	70.4±14.0	64.2±11.3
Early menopause	No^A^	62.7±16.3	65.4±14.3	69.8±18.9	63.1±13.3
	Yes^B^	63.3±13.4	68.6±13.7	65.6±20.1	65.9±11.5
Vitamin D deficiency	No^A^	66.1±16.4	69.4±14.1^B^	73.2±18^B^	67±12.2^B^
	Yes^B^	61.3±15	64.5±14	66.7±19.5	62.2±13
Long-term hormone therapy	No^A^	63.3±15.3	66.6±14^B^	69.8±17.8^B^	63.9±12.8
	Yes^B^	52.5±19.7	54.2±14.9	42.7±34.5	61.5±17.2
Visual impairment	No^A^	62.8±15.4	65.6±14.8	66.9±19.4	63.7±13.5
	Yes^B^	63.1±16.2	67.3±12.5	73.2±18.2	64±11.4
Little or no physical activity	No^A^	65.3±15^B^	67.9±13.7^B^	70±19.4	65.4±12.8^B^
	Yes^B^	51.3±13.1	57.7±13.6	63.2±17.7	55.7±10.4
Previous osteoporotic fracture	No^A^	63.5±14.4	66.2±14.1	68.7±19.6	63.9±12.9
	Yes^B^	56.4±24.8	65.7±14.8	69.6±15	62.1±13.4

Tables 4, 5 display multiple linear regression-identified factors independently associated with quality-of-life domains in women with osteoporosis after adjusting for confounders. Older age was associated with poorer physical and psychological health (p<0.05). Physical inactivity had a negative relation with physical, psychological, and environmental domains (p<0.001). Long-term hormone therapy is related to lower scores for physical, psychological, and social functioning (p<0.05). Higher family income predicted better psychological health and environmental quality of life (p<0.05). Employment positively affected social relationships and the environment (p<0.05). Hyperthyroidism is correlated with better social functioning (p<0.001). Malabsorption syndrome was linked to poorer social relations (p = 0.002). Bisphosphonate use was associated with improved environmental domains for participants (p = 0.042). Overall, the study identified several factors independently associated with quality of life in women with osteoporosis. Modifiable factors, including physical activity, hormone therapy, employment status, and bisphosphonate medication use, emerged as significant determinants of quality of life in osteoporotic women after controlling for confounders. Targeting these factors may help improve well-being in this high-risk population.

**Table 4:** Regression analyses for 26-item dimensions were adjusted for significant confounders for the physical and psychological health domains. Multilevel regression analysis, CI: confidence interval. Family income*: 1: less than 3000, 2: 3000–5000, 3: 5001–10,000, 4: 10,001–20,000, and 5: More than 20,000.

Questions (items)	Physical health				Psychological health			
	Beta	95% CI		P-value	Beta	95% CI		P-value
Age category	−0.24	−10.9	−2.8	0.001	−0.16	−7.9	−0.5	0.028
Little or no physical activity	−0.33	−19.6	−7.7	0.000	−0.29	−16.5	−5.5	0.000
Long-term hormone therapy	−0.18	−26.2	−2.9	0.015	−0.22	−27.2	−5.8	0.003
Family income^*^	−0.08	−4.0	1.4	0.351	0.21	0.9	4.9	0.006

**Table 5:** Regression analyses for 26-item dimensions were adjusted for significant confounders in social relations and environment. Multilevel regression analysis, CI: confidence interval. Family income*: 1: Less than 3000, 2: 3000–5000 3: 5001–10,000, 4: 10,001–20,000, 5: More than 20,000.

Questions (items)	Social relations				Environment			
	Beta	95% CI		P-value	Beta	95% CI		P-value
Little or no physical activity	−0.12	−15.2	3.4	0.211	−0.28	−14.5	−4.4	0.000
Long-term hormone therapy	−0.40	−55.2	−24	0.000	−0.06	−16.2	8.0	0.227
Family income*	0.05	−2.2	4.4	0.586	0.22	0.9	4.6	0.004
Employment status	0.15	0.3	6.8	0.034	0.22	0.4	5.1	0.022
Hyperthyroidism	0.32	13.1	42.9	0.000	0.05	−19.8	7.3	0.601
Malabsorption syndrome	−0.25	−45.4	−10	0.002	−0.09	−19.8	7.3	0.360
Visual impairment	0.15	0.3	12.3	0.041	0.02	−4.4	5.2	0.868
Deficiency in dietary calcium intake	−0.04	−9.3	5.5	0.605	−0.16	−6.3	−0.4	0.035
Bisphosphonates	0.04	−18.9	31.0	0.633	0.15	0.6	28.1	0.042

## Discussion

This cross-sectional study offers essential insights into the ramifications of osteoporosis and fragility fractures across various HRQoL domains in post-menopausal women in the Jazan region.

The research shows a significant distribution of osteoporotic fractures among the studied cohort. Foot fractures emerged as the most common, accounting for 35% of fractures, while hand fractures followed closely at 31%. Previous research suggests that distal fractures are predominantly prevalent in post-menopausal women [16,17]. This highlights a noteworthy frequency of fractures in the extremities, potentially interconnected with stability and fall-related factors. Especially among older adults with osteoporosis, the propensity for falls is elevated, often attributed to compromised balance and muscle strength. Since the foot is integral to maintaining postural stability, it becomes particularly vulnerable to injuries and fractures following a fall. Hand fractures, on the other hand, may result from an instinctive urge during a fall to brace oneself, thereby subjecting the hands to impact. Proactive measures such as screening for potential fall risks and multifaceted interventions encompassing strength training are advocated to avoid such fractures [18,19].

In contrast, the principal weight-bearing bones of the femur and vertebrae constituted merely 11% and 10% of fractures, respectively, despite vertebral fractures being associated with chronic pain, disability, and a deteriorated quality of life [20,21]. Global research consistently echoes our findings, underscoring the profound detrimental effects of vertebral fractures on quality of life across diverse cultural contexts [22-24]. The divergence between the high prevalence of extremity fractures and the relatively low incidence in weight-bearing bones may be explained by the structural robustness and anatomical morphology of these bones, which are inherently adapted to endure weight and stress. However, despite their lower prevalence, the consequences of femur and vertebral fractures can be debilitating, often curtailing mobility and quality of life. Moreover, the conspicuous low incidence of hip fractures, representing only 5% of cases, beckons further scrutiny, considering that hip fractures are conventionally among the most common and severe osteoporotic fractures, subsequently leading to increased mortality and diminished functionality in the elderly population [25]. In the USA alone, as many as 1.5 million osteoporotic fractures, including 250,000 hip fractures, are recorded annually [25]. A past study found that the mortality rates for patients with hip fractures are considerably high, ranging from 15% to 36% [26].

Socioeconomic factors, including unemployment, restrained education, and financial adversity, are significantly correlated with diminished functioning across HRQoL domains, consonant with existing literature illustrating the intricate relationships between socioeconomic disadvantage and well-being [27,28]. Factors such as pervasive feelings of despondency and restricted access to healthcare services might underpin these observations. Initiatives to bolster educational and vocational avenues for women facing socioeconomic challenges could lessen their quality of life. Interestingly, married women had lower social functioning, contrasting Western trends [20]. This discrepancy can be attributed to stringent gender norms that curtail the social agency of married Saudi women [29,30].

Regarding physiological conditions, the investigation revealed a discernible decline in HRQoL, particularly in physical health, with advancing age, possibly reflecting an incremental deterioration of physical ability and the onset of comorbidities with aging [31]. Furthermore, the relationship between chronic conditions and HRQoL is unequivocally affirmed, as chronic diseases invariably impinge upon HRQoL via physical limitations, psychological coercion, and the inherent burden of disease management [32,33]. While hypertension may ostensibly remain asymptomatic, the management and complications thereof can impair HRQoL, as evidenced by existing literature delineating its adverse effects on physical activity and health perceptions [34,35]. Thus, a multifaceted strategy beyond mere blood pressure control may be required. Likewise, RA intuitively reduces physical HRQoL, reflecting its characteristically debilitating effects on physical function and pain. Our study also unearthed that bisphosphonate administration was concomitant with enhanced psychological HRQoL, underscoring their efficacy in augmenting quality of life through the prevention of fractures [36,37]. Conversely, prolonged hormone therapy was discerned to adversely affect several domains, a finding that accentuates the associated risks delineated in previous research [38,39]. Modifiable risk factors, including vitamin D insufficiency and sedentary lifestyles, surfaced as pivotal determinants to address via targeted interventions [22,24,38].

Regression analysis revealed several noteworthy relationships in examining the factors associated with HRQoL. Advanced age and limited physical activity were negatively associated with physical and psychological health, underscoring the need for age-specific interventions and physical activity promotion across demographics. Previous research reported that physical activity declines with age in post-menopausal women and affects HRQoL [40]. A study found that post-menopausal women who met public health physical activity guidelines reported higher HRQoL on both the physical and mental components than women not achieving these guidelines [41].

Long-term hormone therapy also demonstrated negative links to physical and psychological health. In contrast, family income and employment positively relate to domains like psychological health, social relations, and environment, highlighting their pivotal roles in well-being through financial stability and social connections. Prior studies corroborate the quality-of-life benefits of higher socioeconomic status and employment. Both higher socioeconomic status and employment, particularly employment of good psychosocial quality, are associated with improved quality of life. However, the relationship between these factors and quality of life is complex and influenced by various other factors, including social capital, lifestyle choices, and the nature of the work [42,43].

Certain conditions like hyperthyroidism, malabsorption syndrome, and visual impairment showed varied associations, necessitating further exploration into the mechanisms influencing these connections. For instance, hyperthyroidism’s link to social relations and malabsorption syndrome’s impact on the same domain likely stem from different root causes like symptoms and dietary restrictions. Meanwhile, visual impairment’s counterintuitive positive association suggests probing supportive factors and coping mechanisms individuals might employ. Additionally, dietary calcium deficiency’s negative tie to the environment indicates nuanced interplays needing illumination. Finally, bisphosphonates’ association with an improved environment potentially reflects their efficacy in osteoporosis management and environmental interaction.

This extensive exploration of HRQoL in the context of osteoporosis among post-menopausal women in the Jazan region reveals imperative insights, simultaneously prompting recommendations for the institution of systematic osteoporosis screening programs, especially targeting those aged 50 and above. Moreover, it underscores the criticality of emphasizing strength training and fall-risk assessments. Public health initiatives, precisely calibrated to cultural sensitivities, should prioritize osteoporosis awareness while concurrently addressing modifiable risk factors such as vitamin D deficiency and physical inactivity. This fosters a nexus between robust scientific research and tangible, impactful public health initiatives, ensuring that scientific findings are seamlessly translated into viable, productive health strategies.

We had some limitations in our study. The cross-sectional design assessed associations at a one-time point, limiting causal inferences. Though powered for analyses, the modest sample size may have precluded detecting smaller effects. Participants were recruited from select clinics, which may limit generalizability. Self-reported data could be subject to recall bias. Assessments did not include objective measures like BMD testing. Given the complex etiology of osteoporosis, residual confounding is possible. The strengths include a community sample, rigorous analyses, and a multidomain HRQoL evaluation. Further longitudinal studies with larger samples could enhance the evidence on how osteoporosis and resultant fractures impact the quality of life in this population.

## Conclusions

This study provides novel evidence that osteoporosis and resultant fragility fractures substantially reduce the physical, psychological, social, and environmental domains of HRQoL in post-menopausal Saudi women. Vertebral and hand fractures were most common and linked with poorer functioning. Advanced age, low socioeconomic status, unemployment, chronic diseases, and modifiable factors like physical inactivity and long-term hormone therapy are negatively related to the quality of life. These results underscore the need for more extraordinary clinical and public health efforts to prevent, screen, and manage osteoporosis in this population. Optimal management of modifiable risks could help mitigate decreases in quality of life. Saudi women of advanced age, low socioeconomic status, or those with vertebral or hand fractures represent high-risk groups who may benefit from targeted, multidomain interventions to improve functioning and well-being. Further research should explore locally validated quality-of-life instruments and assess impacts in larger samples with objective clinical data.
